# Patients’ views on the decision to investigate cancer symptoms in older adults: a qualitative interview study in primary care

**DOI:** 10.3399/BJGP.2022.0622

**Published:** 2023-06-27

**Authors:** Daniel Jones, Omer M Ali, Stephanie Honey, Claire Surr, Suzanne Scott, Niek De Wit, Richard D Neal

**Affiliations:** Leeds Institute of Health Sciences, University of Leeds, Leeds, UK.; Leeds Institute of Health Sciences, University of Leeds, Leeds, UK.; Leeds Institute of Health Sciences, University of Leeds, Leeds, UK.; Leeds Beckett University, Leeds, UK.; Wolfson Institute of Population Health, Queen Mary University of London, London, UK.; Julius Center for Health Sciences and Primary Care, and professor of general practice, University Medical Center, Utrecht, the Netherlands.; Department of Health and Community Sciences, Faculty of Health and Life Sciences, University of Exeter, Exeter, UK.

**Keywords:** frailty, investigation, neoplasms, older adults, primary health care

## Abstract

**Background:**

Cancer is predominantly a disease of older adults. To date there has been little research on the experiences of older adults or their views on the diagnostic pathway.

**Aim:**

To gain an improved understanding of the views and experiences of older adults on all aspects of cancer investigation.

**Design and setting:**

This was a qualitative study using semi-structured interviews with patients aged ≥70 years. Patients were recruited from primary care in West Yorkshire, UK.

**Method:**

Data were analysed using a thematic framework analysis.

**Results:**

The themes identified in participants’ accounts included the patients’ process of decision making, the value of having a diagnosis, the patients’ experience of cancer investigations, and the impact of the COVID-19 pandemic on the diagnostic pathway. Older adults in this study indicated a clear preference for having clarity on the cause of symptoms and the diagnosis, even in the face of unpleasant investigations. Patients suggested they wanted to be involved in the decision process.

**Conclusion:**

Older adults who present to primary care with symptoms suggestive of cancer may accept diagnostic testing solely for the benefit of knowing the diagnosis. There was a clear patient preference that referrals and investigations for cancer symptoms should not be deferred or delayed based on age or subjective assessments of frailty. Shared decision making and being involved in the decision-making process are important to patients, regardless of age.

## INTRODUCTION

Worldwide, the population of adults aged >70 years is growing faster than any other age group.^1^ The burden of cancer falls predominantly on older patients with half of all new diagnoses occurring in people aged >70 years and incidence rates for all cancers increasing most rapidly in the >75 years age group.^1^^,^^2^

Diagnosing cancer at an early stage is important and associated with improved survival.^3^ However, in older adults, these survival benefits are likely to be reduced, because of shorter life expectancy. If cancer is diagnosed, older and frail patients have an increased risk of morbidity and mortality from cancer surgery and intolerance of chemotherapy and radiotherapy.^4^ Decisions to investigate or refer older adults with cancer symptoms may also be complicated by the presence of cognitive impairment, frailty, and comorbidities.^5^^,^^6^ In older adults there is a different health-related context and the decision-making process is different. Prognosis is reduced, and there are increased risks of treatment side effects. This requires well-balanced decisions in which the individual perspective is taken into account. National Institute for Health and Care Excellence (NICE) guidelines for suspected cancer (NG12) outline possible symptoms of cancer and the appropriate investigations and referrals that should be arranged.^7^ Although these guidelines suggest shared decision making, there is no specific guidance on how to implement this in the management of older adults or those with frailty.^4^ Frailty has been defined by Clegg *et al* as a state of increased vulnerability to poor resolution of homoeostasis after a stressor event, which increases the risk of adverse outcomes, including falls, delirium, and disability.^8^

To the authors’ knowledge, no research to date has considered the value of a diagnosis as perceived by older adults nor their views on the need for shared decision making in cancer care. This qualitative study aimed to gain an insight into the views and experiences of older adults who presented to primary care with symptoms that may be caused by cancer.

## METHOD

### Study design

The study took a qualitative approach using semi-structured telephone interviews to assess patients’ perceptions of the factors that influence the decision to investigate cancer symptoms in older adults, the value of having a confirmed diagnosis, and the experiences of, and preference for, shared decision making. Telephone interviews were chosen for patient safety during the COVID-19 pandemic.

**Table:** How this fits in

The decision to investigate and refer older adults with symptoms suggestive of cancer can be complicated by frailty and comorbidities. Treatment options for older adults diagnosed with cancer can be limited because of frailty and reduced life expectancy. This interview study found that older adults supported early referral and investigation of cancer symptoms even in the face of invasive tests. The older adults interviewed showed a preference for shared decision making before referral.

### Definition of older adults

Frailty and comorbidities increase with increasing age.^9^ The aim of the study was to investigate how older age and frailty has an impact on patients’ preference for cancer investigations. The definition of older adults used was >70 years old, following an Office for National Statistics report on ageing based on the latest UK census.^10^

### Sampling and recruitment

Participants aged ≥70 years who had presented to general practice with a symptom suggestive of cancer or had been referred on an urgent cancer referral within the past 6 months (as listed in NICE NG12 cancer guidelines) were included. Any patient with signs of cognitive impairment or dementia were excluded if their GP felt they could not consent. Patients were also excluded if the possibility of cancer had not been discussed in their GP consultation or if they did not speak English. Participants were recruited from six general practices in West Yorkshire, selected via the local clinical research network. West Yorkshire is ethnically and socioeconomically diverse and is representative of the UK population. Patients were identified through searches of general practice electronic patient records for patients who met the inclusion criteria. Potentially eligible patients were screened by their GP to ensure they met the criteria. These patients were then contacted by the practice via a letter explaining the study and inviting potential participants to contact the research team. The research team then explained the study and sent an information sheet and the consent form via post or email. Participants were entered into the study once the consent form had been signed and returned to the research team.

Sample size was determined using guidelines by Francis *et al.*^11^ An initial 10 interviews were carried out and analysed. Following this, three further interviews were conducted and analysed; this continued until there were three consecutive interviews without additional themes being identified.

### Data collection

The telephone semi-structured interviews were carried out between January and July 2022 by an experienced qualitative researcher (one of the authors). The interview followed a topic guide that was developed from previous research on experiences of cancer decision making,^12^ and in consultation with the researchers’ patient and public involvement group. Participants were asked to describe their most recent experience of presenting to general practice with symptoms and the process of investigations and referrals that followed. Participants were asked about their involvement in the decisions made, and their preferences for shared decision making. The topic guide also contained sections exploring participants’ reasons for opting to investigate symptoms and what impact their own health had on the decisions made. During the interviews, audio data were recorded. The interviews were transcribed verbatim and anonymised.

### Analysis

Analysis of the data was undertaken using framework thematic analysis. The analysis consisted of five steps documented by Srivastava *et al*, which include familiarisation, identifying a thematic framework, indexing, charting, and mapping and interpretation.^13^

During familiarisation, two of the authors independently immersed themselves in the data through reading and re-reading all transcripts while making notes on key ideas and recurrent themes. Once the researchers had become familiar with the data, the next step was to identify a thematic framework. This consisted of developing the key issues and concepts. This was carried out inductively rather than having a priori ideas. The two researchers worked independently and then came together to discuss and agree on a thematic framework. The thematic framework was then applied to each transcript used to index the data, line-by-line, using NVivo version 12. NVivo was used to facilitate organisation and allow retrieval of the indexed data. To ensure consistency, the two researchers initially indexed four transcripts (approximately 20%), at which point indexing by each researcher was compared and differences were discussed. For example, barriers to shared decision making had been indexed with differing frequency; following discussion, the authors agreed on examples of barriers. The remaining transcripts were then indexed by both researchers.

Charting was used to gain a better understanding of the data as a whole. Charting involved rearranging the data in a matrix with subheadings from the thematic framework and participants’ indexed data that best corresponded to each theme. Once the data had been charted according to the main themes, the final stage of mapping and interpretation was undertaken. This stage consisted of analysing the data by comparing and contrasting different experiences and perceptions, looking for similarities, and linking this back to the original research question.

## RESULTS

A total of 18 participants were recruited from six different GP practices across rural and urban areas of West Yorkshire. The participants were aged between 73 years and 88 years with a mean age of 81 years. All the participants identified as White British and seven were female. Five participants lived alone; 13 lived with their spouse. Six of the participants had higher-level degrees, four had O-levels, and the remaining eight had no qualifications.

Participants discussed their perception of the process of decision making and the need to be involved, the value of having a diagnosis, their experience of cancer investigations, and the impact of the COVID-19 pandemic on the diagnostic pathway.

### Importance of shared decision making

The participants discussed the process of reaching a decision with the GP to investigate cancer symptoms. Although showing a preference for shared decision making, participants often indicated that they would follow the advice of the doctor:

*‘Oh yes, I was involved, and he explained to me what it entailed and I was happy to just go along with things.’*

(Participant [P] 9, Male [M], aged 80 years)

*‘Well, I just took the advice of the doctor. He gave me choices and he might think, “So and so”, but he did not say, “You must do this” and I usually agree with him because he is the man* [laughter]. *He knows more than I ever will, so …’*

(P13, M, aged 88 years)

Other participants were clear that they felt it was important that they were involved in decision making about potential cancer diagnostic investigations, and some gave examples of being given a clear choice to make:

*‘Once there, he said, “Well, we can do one of three things.” He said, “We can forget about it altogether and just let it go and see how it goes or we can have a test, one of the scan things, or we can have one down your mouth, down your throat and into your stomach” and I did not fancy that at all, so I went for the middle one. Anyway, so I was really involved in that.’*

(P3, M, aged 85 years)

*‘I’m old, but I’m also quite intelligent. I need to know what’s going on. I’m not happy with being kept in the dark about anything. I think it’s my body, and it’s my decisions as to what I do. Obviously, I’ve got to be involved in the decision making.’*

(P6, Female (F), aged 82 years)

On the other hand, a minority of participants gave examples that suggested the GP had made a decision on their behalf:

*‘I explained what my concern was and we discussed it in a simple way and he said, “Right, we’ll have to investigate”, and he set up the necessary things and blood tests and a head scan. And that’s all gone ahead, and I’m waiting for results now.’*

(P5, M, aged 78 years)

*‘He sent through the PSA* [prostate-specific antigen] *test results and said I really think that you ought to go for a biopsy and everything else that goes with it.’*

(P2, M, aged 83 years)

The participants discussed barriers to shared decision making and suggested ways in which it could be improved. Lack of continuity of care, lack of time within the consultation, and the patient’s perception that the doctors were busy or overstretched were all highlighted as barriers to shared decision making:

*‘As with all general practices now, every time you go you see a different GP. So there’s no continuation. You have to start again every time and there’s no relationship, if that’s the right word, built up between the patient and doctor.’*

(P5, M, aged 78 years)

*‘They are so busy. You are in and out and it is all … it is not like it used to be in the old days when the GP used to sit down … now, it is all very automatic … I suppose they just need a bit more time and also perhaps a slightly more personal touch. I don’t know.’*

(P12, F, aged 82 years)

Participants also highlighted communication difficulties related to hearing loss that could impact shared decision making:

*‘When you walk into the surgery to meet the doctor, you shuffle in on a Zimmer frame or whatever and you can hardly talk or hear what they are saying.’*

(P2, M, aged 83 years)

### Patients’ experience of cancer investigations

Patients described their negative experience of having investigations for possible cancer, using terms such as traumatic, painful, unpleasant, and in one case ‘absolutely horrific’:

*‘It was rather rigorous. Because I don’t know whether you have heard of it. I’ve had a relative who has had it. Empty your system overnight which is rather traumatic. Yes, it was, it was quite painful. I did ask for the sedative which they gave me, but it didn’t knock me out because they wanted me to cooperate with movement, you see.’*

(P2, M, aged 83 years)

*‘I had an endoscopy. That was awful. I didn’t care for that. It was the tube down the throat that I didn’t care for.’*

(P9, M, aged 80 years)

Despite the participants’ negative experience of tests there was an acceptance that, if necessary, they would have the tests again, such was the importance of having clarity on the diagnosis:

*‘If I have to have an endoscopy again, I would just go along with it because I can see that it’s necessary to find out what’s going on in your body.’*

(P9, M, aged 80 years)

*‘Some of the machines that they put me through for the prostate were absolutely horrific, but I’d do it again if I thought I had to.’*

(P18, M, aged 81 years)

### Value of a diagnosis

Participants discussed the importance of knowing the cause of their symptoms. Participants considered a confirmed diagnosis to be essential:

*‘I’d rather know. Yes, I’d rather know. Oh I’d have the tests if it meant, you know. I’d have the tests. Whatever advice they have got, I’d give it a try.’*

(P4, F, aged 78 years)

*‘There is no point in going and seeing a doctor if you do not want to know what is going on.’*

(P11, M, aged 81 years)

Participants gave multiple reasons for placing such a high value on knowing the diagnosis. These included being able to act on the diagnosis, either by making lifestyle changes or considering new medications or treatments:

*‘If your car goes wrong, you want to know what’s wrong with it. Well, you have to know what’s wrong with it before they can mend it. And I feel that’s very much the same with your body. If something goes wrong, find out what it is, and then put it right if you can.’*

(P5, M, aged 78 years)

*‘If you don’t know, you can’t do anything about it, can you? I didn’t know whether it was diet or there was something wrong in my body that I had some control over.’*

(P9, M, aged 80 years)

Others felt having clarity over the diagnosis would alleviate fear or worry over the cause of the symptoms:

*‘Well, because if you can understand what is going on, then it is not so … if you do not know what is causing something, then you can worry about it more, can’t you?’*

(P12, F, aged 82 years)

### Impact of increasing age and frailty on decisions to investigate symptoms

Some participants discussed if there would be a time when they would not wish to investigate possible cancer symptoms. During these discussions, the value of a diagnosis and need for possible treatment was thought to be less important in those who were very old and frail. However, the participants suggested that at present they were not too old for investigations and treatment:

*‘There must be a time when you think, “I have just had enough of this lot. I will just soldier on and when I die, I die”, but I have not reached that stage yet at all* [laughter]. *I think if I get really old and rickety, there must be a time when you say, “Enough is enough”, but I am nowhere near that yet, I hope.’*

(P13, M, aged 88 years)

### Impact of the COVID-19 pandemic

The impact of the COVID-19 pandemic was noted in the conversations with participants. The COVID-19 pandemic affected the participants in a number of different ways. The main impact that was noted by the majority of participants was the increased use of technology and the difficulty of having a face-to-face appointment. This was universally disliked by the older adults:

*‘The thing is it is getting to see a GP these days because everything seems to be wanting to be done on the telephone and I do not think it is a very good situation. There are a lot of people, particularly my age anyway, not so much the young ones, but you like to have a discussion eyeball to eyeball.’*

(P11, M, aged 81 years)

There were also some examples of the pandemic resulting in patients delaying their presentation to primary care:

*‘I’m not up on technology at all. I’m not, I’m hopeless at it. I can just about go on Zoom for meetings. And it sounded so complicated and so much, I didn’t want to do it.’*

(P4, F, aged 78 years)

*‘I would have gone eventually, but as it was lockdown, I did not like to. I thought it was too trivial to bother with really.’*

(P12, F, aged 82 years)

## DISCUSSION

### Summary

To the authors’ knowledge, this is the first qualitative study to consider the decision-making process regarding investigations and referral of cancer symptoms in older adults. The results are comparable with a recent systematic review that found frailty, comorbidities, involvement of family and carers in decision making, and consultation time affect the decision to investigate potential cancer symptoms.^14^

The value of a diagnosis is something that has been the subject of debate. Although it is recognised that an accurate and timely diagnosis allows the best health outcomes for patients by allowing tailored clinical decision making^15^ there is an acknowledgement that diagnostic uncertainty is inevitable and ‘overly aggressive diagnostics’ may put patients at greater risk of harm, without improving outcomes.^16^ The key factor emphasised was the high value participants placed on having a diagnosis that could explain their symptoms, provide a possibility of self-management, as well as give an indication of what is to come. The perceived importance of confirming a diagnosis at any price was apparent despite the fact that most participants described having a negative experience of cancer investigations.

This study found a clear preference for diagnostic testing even in old age. Although patients seemed to show a preference for being involved in decisions around their care, most suggested that they would follow the advice of the professionals. A number of barriers to shared decision making were identified by the participants, which included a lack of continuity of care, short consultation times, and communication difficulties.

The changes to primary care as a result of the COVID pandemic were unanimously negative according to the participants. The decrease in face-to-face appointments and the increased use of technology and telephone consultations was a key change identified by participants.

### Strengths and limitations

This qualitative study followed a structured and widely recognised method of analysis and is in line with reporting guidelines for qualitative research.^17^ The use of semi-structured interviews ensured key questions were asked to all participants. By being semi-structured it allowed the interviewer to probe answers that were significant or ambiguous for further information and clarification. The demographic characteristics were diverse in key areas, the study had a mix of males and females, the participants came from a mixed educational background as well as having a mix of previous occupations.

This study did not compare responses according to gender or other sociodemographic characteristics and this would be useful to investigate in future quantitative research. All the participants interviewed in the study had presented to their GP with symptoms during a COVID-19 lockdown. It is possible that this sample is a select group, in whom a diagnosis was important. Similarly, the method of recruitment, which required participants to contact a research team, could result in a biased sample. None of the sample were diagnosed with cancer following their presentation, therefore this has not captured the views of anyone who potentially received a cancer diagnosis. All the participants identified as White British; as a result the study does not include the views of individuals from different ethnicities. The study did not take into account the individual’s historical perspective, such as experiences with medical investigations, with health decisions in person, or in the family, or the patients’ level of frailty. Excluding patients who were unable to give informed consent meant that patients with cognitive impairment were not interviewed, potentially excluding an important group of older adults with potentially challenging decisions regarding referral and investigation of cancer symptoms.

The use of telephone interviews was necessary because of the COVID-19 pandemic, but it resulted in an inability to assess the non-verbal cues when interviewing a participant. Further to this, the limitation was compounded by the older age of the participants: the older participants stated they disliked telephone appointments with their GPs and preferred face-to-face appointments, with one participant stating they were hopeless with technology. However, the study was designed and conducted during the COVID-19 pandemic and as a result telephone interviews were the only viable option available at the time.

### Comparison with existing literature

This study found a clear preference for patients to accept diagnostic testing even in old age. This is supported by a vignette study that explored participant preferences for diagnostic testing.^18^

The current study’s mixed finding of older adults’ desire, or not, to be involved in decision making was supported by a qualitative study conducted by Butterworth and Campbell,^19^ who considered older adults’ perceptions of shared decision making in general practice and reported patients having a *‘spectrum of involvement’* in decisions, from those *‘overwhelmed by the complexity of information’*, to others who *‘had taken a decision into their own hands’*.

A number of barriers to shared decision making were identified by the participants. This is in line with a study that focused on shared decision making in older people with multimorbidity that highlighted a number of barriers to shared decision making including a lack of time within the consultation and lack of continuity of care.^20^ Similarly, a review of studies aiming to support shared decision making in older adults highlighted the benefit of face-to-face interactions and continuity of patient–professional relationships.^21^

A report by Cancer Research UK identified challenges of engaging in shared decision making with older people.^9^ The main challenge was that older people were less likely to question a doctor, preferring to simply follow their advice, which was also described within the current study. The report also identified that treatment decisions for older patients are likely to be more complex. This is because of older people having to weigh up their quality of life against length of life when considering decisions about cancer investigations and management.^9^ Although the majority of participants were willing to have invasive investigations, they recognised that as they became older and frailer there may be a time when they would not want to be investigated, suggesting that they may prefer to ‘go quietly’ or say ‘enough is enough’. However, despite the mean age of the group being 81 years old, most participants felt they personally were not yet old enough to refuse investigations yet, but may in the future.

The changes to primary care because of the COVID-19 pandemic were unanimously negative according to the participants. The decrease in face-to-face appointments and the increased use of technology and telephone consultations were key changes identified by participants.

### Implications for research and practice

This study suggests that older adults who present to a GP with symptoms that may be indicative of cancer can have a clear preference for knowing the diagnosis, even in the face of unpleasant investigations. Patients felt knowing that the diagnosis may allow for self-management and could alleviate worry or fear of the unknown. However, the authors recognise this research was solely based in West Yorkshire, lacked ethnic diversity, and did not include those with cognitive impairment; as such, the findings may not be transferable to all populations served within UK general practice.

The current NICE guidelines on the recognition and referral of cancer symptoms support the findings, providing no upper age limits for cancer investigations or referrals but suggesting the importance of shared decision making.^7^ However, a report by Cancer Research UK suggested that there is a danger of overestimating frailty in general practice with assessments based on assumptions, rather than established frailty scores, and this may result in patients not being referred for cancer tests. Data from the current study suggest that older adults would accept diagnostic testing solely for the benefit of knowing the diagnosis and as such there is a clear patient preference that referrals and investigations for cancer symptoms should not be deferred or delayed based on age or subjective assessments of frailty.^8^ This concept of ‘needing to know’ may be at odds with other guidance within general practice, where concerns about overdiagnosis and overinvestigation have steered GPs away from actions that may not change patient treatment or outcomes.^22^ For example, this study would suggest that even a patient who has indicated that they would refuse treatment in the form of surgery, chemotherapy, or radiotherapy for any potential cancer may still benefit from diagnostic testing in order to know their diagnosis and to support advanced care planning.

Patients in the study were supportive of shared decision making and, although most felt that they would be guided by the professional, they also wanted to feel involved in the process. This seemed conflicting: why advocate shared decision making, to then follow the doctor’s advice? Do patients solely want a clear explanation of all available options? Or do they value the ability to make their own decisions in some circumstances? Despite this contradiction, the NHS Long Term Plan aims for patients to have a *‘shared responsibility for health’* by providing support for patients to manage their own health. GPs could facilitate this by explaining not only the benefits, but also the potential harms and side effects of investigations.^23^

This current study identified a number of barriers to shared decision making in primary care. The barriers identified included short consultation time, lack of continuity of care, and the need for family members to be present in the consultation. Further work is required in primary care to address these barriers. This will involve identification of patients who are frail, which could potentially be done using prediagnostic frailty scoring systems. Patients identified as frail could have arrangements put in place to ensure best possible care, such as providing face-to-face appointments and encouraging family members to be present during the appointment if they desire. Further to this, it could allow longer appointments for these patients to ensure shared decision making is possible. There also could be an attempt to ensure the patient is seen by a named GP to improve continuity of care.

Finally, patients did suggest that there may come a time where cancer investigations may not be in their best interests. Research has shown that older and frail patients have an increased risk of morbidity and mortality from cancer surgery, and intolerance of chemotherapy and radiotherapy.^4^ Two studies focusing on the impact of comorbid cancer and dementia on decision making suggest the process is challenging and requires careful and ongoing consultation, with participants showing a preference for quality of life over life expectancy.^12^^,^^24^ As a result, further guidance is required to assist GPs in managing patients who are severely frail and older, and who have cancer symptoms. Support is needed to encourage the symptomatic management of these patients, as well as signposting to other support and services that could be provided to assist these individuals who may have cancer.

## Data

Participants did not give consent to share their interview transcripts beyond the immediate research team.
